# Metachronous Bilateral Testicular Plasmacytoma After an Initial Soft Tissue, Extramedullary Plasmacytoma

**DOI:** 10.7759/cureus.19517

**Published:** 2021-11-12

**Authors:** Ioannis Manolitsis, Lazaros Tzelves, Vassilis Koutoulidis, Maria Gavriatopoulou, Ioannis Varkarakis

**Affiliations:** 1 Second Department of Urology, National and Kapodistrian University of Athens, Sismanogleio General Hospital, Athens, GRC; 2 First Department of Radiology, National and Kapodistrian University of Athens, Aretaieion Hospital, Athens, GRC; 3 Department of Clinical Therapeutics, School of Medicine, National and Kapodistrian University of Athens, Greece, Alexandra General Hospital, Athens, GRC

**Keywords:** testicular neoplasms, extramedullary multiple myeloma, testicular mass, testicular plasmacytoma, testis

## Abstract

Testicular plasmacytoma is a rare extramedullary manifestation of plasma cell dyscrasia. We report a case of a 53-year-old man who presented with a metachronous testicular lesion originating from a soft tissue plasmacytoma of the oral cavity. He underwent radical orchiectomy and a year later, presented with testicular plasmacytoma in the contralateral testis. He received induction chemotherapy, autologous stem cell transplantation with high dose melphalan and maintenance with lenalidomide, as he refused orchiectomy and achieved complete remission. However, six months post-transplant, he relapsed with localized disease in his testis, received second-line chemotherapy, after refusing orchiectomy and still remains in partial remission and alive.

## Introduction

Multiple myeloma is a malignant disease characterized by neoplastic proliferation of monoclonal plasma cells inside the bone marrow. Multiple myeloma accounts for approximately 10% of all hematological malignancies and 2% of all newly diagnosed cancers, affecting mainly the elderly population. The diagnostic criteria of multiple myeloma include the presence of at least 10% abnormal plasma cells in the bone marrow or histologic proof of a plasmacytoma, clinical features of multiple myeloma, and at least one of the following abnormalities: monoclonal serum protein (usually greater than 3 g/dL), monoclonal protein in the urine, or osteolytic lesions [1]. It has been reported that up to 30% of people diagnosed with multiple myeloma, will present with the extramedullary disease during their disease course, either at initial presentation (6%-8%) or as at the relapsed/ refractory setting (10%-30%) [1,2]. Extramedullary disease comprises three different clinical entities: extramedullary plasmacytomas that are soft tissue tumors or clonal plasma cell infiltrations of anatomical sites distant from the bone marrow through the hematogenous spread, plasmacytomas that are in direct contact with bone structures, and plasma cell leukemia [2,3]. Extramedullary plasmacytoma may infiltrate various anatomic sites with skin, liver and central nervous system being the most frequently involved [4]. Testicular involvement of this disease is a rare clinical entity, with around 70 cases reported in the literature, patients may present with the extramedullary disease either at the time of diagnosis or at disease relapse. [5,6]. We report here a case of testicular plasmacytoma, metachronous to a soft tissue plasmacytoma, that relapsed after surgical treatment, to the contralateral testicle.

## Case presentation

A 53-year-old man presented with a gradually increasing mass located in the soft tissue of the oral cavity. The lesion was biopsied, and the pathology report showed increased infiltration of the buccal mucosa with monoclonal plasma cells. Immunochemistry demonstrated positivity for CD138, CIgA, and negativity for CD56, Cyclin D1 and CD20. Therefore, the diagnosis of soft tissue plasmacytoma was confirmed, for which he received localized radiotherapy with a total dose of 40 Gy, due to the absence of systemic disease as the bone marrow biopsy revealed the absence of neoplastic infiltration and serum and urine immunofixation were all negative.

**Figure 1:**
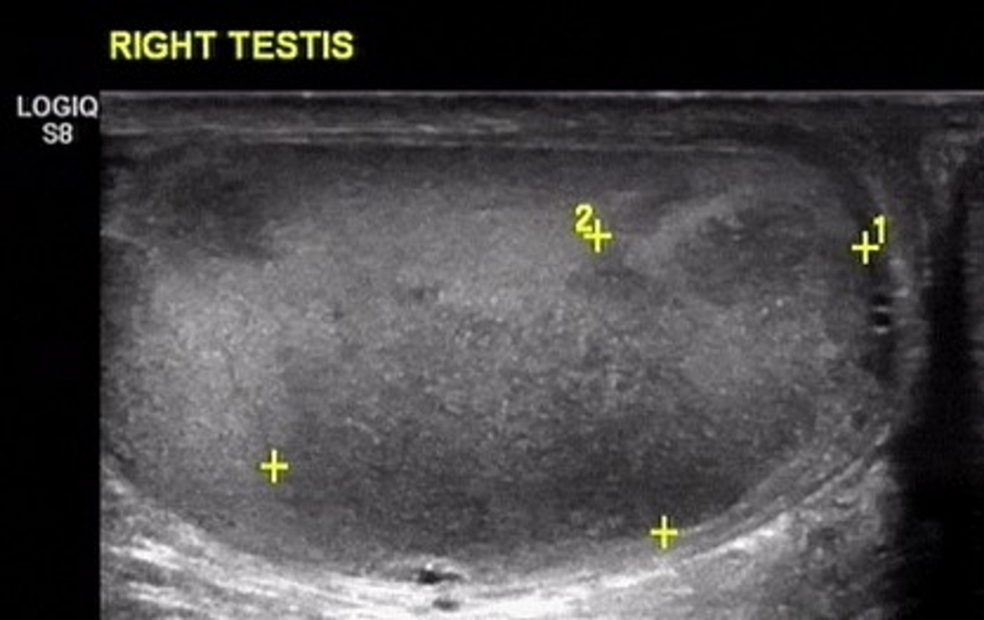
A hypoechoic mass of 1.02 x 0.59 cm seen in the right testis

After five years, the patient noticed a painless swelling in his right testis. An ultrasound of the scrotum was performed that showed a hypoechoic mass in the right testicle with increased vascularization, and a normal-appearing left testis (Figure 1). The patient subsequently underwent a right radical orchiectomy and the histopathology report showed testicular infiltration by a plasma cell neoplasm with identical immunophenotype (CD138+, CIgA+, CD56-, CD20-, Cyclin D1-) to the primary site in the oral cavity. Subsequently, the patient underwent a bone marrow biopsy that showed the absence of monoclonal plasma cell infiltration, and a PET/CT scan that was negative for reactive lesions suspicious of malignancy. In addition, serum and urine protein electrophoresis, as well as serum-free light chain assay were all within normal range, thus, excluding the presence of systemic disease, while complete blood count and full biochemical profile were normal.

**Figure 2:**
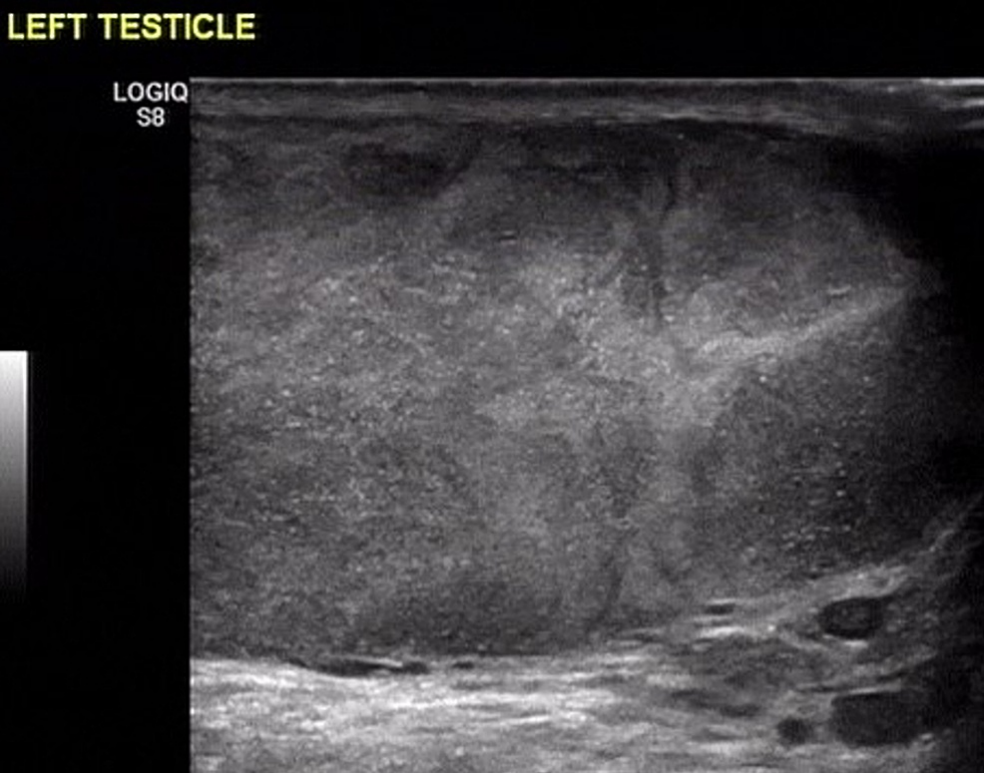
Enlarged left testis with diffuse parenchymal heterogeneity

**Figure 3:**
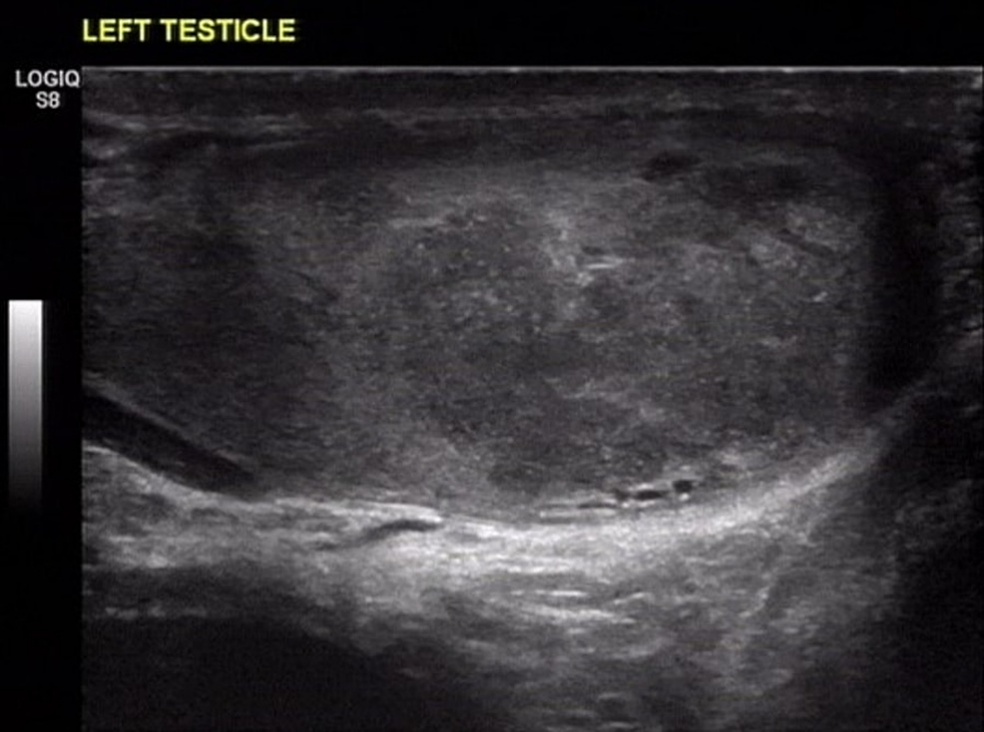
Left testicular parenchyma filled with hypoechoic lesions

**Figure 4:**
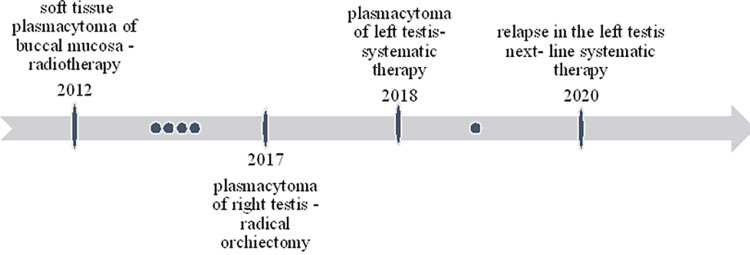
The timeline of the disease

However, after one year, the patient presented again with painless swelling of his left testis that was attributed to disease relapse based on the imaging findings (Figure 2). He refused to undergo left orchiectomy, thus he proceeded with systemic treatment based on lenalidomide, bortezomib and dexamethasone. Following 4 cycles of treatment, the patient underwent high dose melphalan with autologous stem cell transplantation. The blood tests of the patient revealed an increase in serum creatinine (1.3 mg/dl with 1mg/dl baseline level), normal calcium levels and normal complete blood count. Complete response was achieved with negative PET/CT scan and negative marrow minimal residual disease assessment. The patient continued on lenalidomide maintenance; however, six months later he was diagnosed with disease relapse in his left testicle (Figure 3). A PET/CT scan was then performed, and showed a reactive lesion with an increased SUV max of 7 in the left testis, with no other loci suspicious of disease relapse. The blood analysis of the patient revealed no abnormal values. Since the patient refused surgery, he was initiated with next-line therapy, with bortezomib, cyclophosphamide and dexamethasone, he responded partially and still remains in remission (Figure 4), while serum protein electrophoresis, immunofixation and serum-free light chain assay, all remain within normal levels.

## Discussion

Testicular involvement in extramedullary disease constitutes a very rare clinical entity. It accounts for less than 0.1% of all testicular masses, and the average age of onset is 55-60 years of age, with the first case being reported back in 1939 [7]. Testicular plasmacytomas occur, in most cases, in patients with systematic multiple myeloma, either at diagnosis or at relapse [5]. However, they can rarely occur in the absence of an established systemic hematologic malignancy [8], as in our case. It has been documented, that the testes constitute a sanctuary site for hematologic malignancies, as a result of the testicular blood barrier, without the involvement of the bone marrow [9]. Bilateral involvement of the testis is extremely rare, with most cases occurring asynchronously at the relapse setting, with only five cases in the literature reporting bilateral synchronous disease [10].

In our case, surprisingly, there was no evidence of systemic disease or relapse either at diagnosis (oral cavity) or at relapse (testis). Initially, the management of the right testis disease required radical inguinal orchiectomy. Our patient relapsed after surgery in the contralateral testis and refused to undergo orchiectomy. There is evidence that patients with plasmacytoma of the testis could benefit from the combination of chemotherapy with autologous stem cell transplant [11], and there are very few documented cases of testicular plasmacytoma relapse after the implementation of autologous stem cell transplantation [12] as in our patient.The prognosis varies among the different types of the disease, with the presence of extramedullary myeloma at diagnosis, being an adverse prognostic factor, in patients with systematic multiple myeloma [13]. Extramedullary relapse poses as an adverse prognostic factor, particularly in patients suffering from bone-unrelated disease [14]. A review by Anghel et al. estimated that nearly 60% of patients died within 26 months of diagnosis of testicular plasmacytoma, either metastatic or primary, and most of them within a year after radical orchiectomy [5]. The available data suggest that our patient is unique, as he is currently in remission after a second relapse, three years from initial diagnosis, with several available therapeutic options that can be used in case of a future relapse. Therefore, our patient represents a case showing the potential of long-term disease survival, adding to the existing knowledge and literature, regarding the management and prognosis of extramedullary disease.

## Conclusions

Testicular plasmacytoma is a rare form of the extramedullary disease that occurs in most cases in the context of systematic multiple myeloma. The prognosis is considered poor with radical orchiectomy being the treatment of choice. Systematic therapy can be an alternative during the disease course. The available data are limited, and due to the rarity of the disease, there is no specific therapeutic algorithm. This poses the necessity for conducting clinical trials to establish specific guidelines on testicular plasmacytomas and extramedullary disease.
